# Explainable artificial intelligence for predicting mortality in geriatric patients undergoing hip arthroplasty: Machine learning analysis using national health insurance data

**DOI:** 10.1097/MD.0000000000050463

**Published:** 2026-09-18

**Authors:** Hyunyoung Seong, Kwang-Sig Lee, Yumin Choi, Jeonghoon Lee, Taesan Kim, Surim Hong, Hyeon Ju Shin, Ki Hoon Ahn

**Affiliations:** a Department of Anesthesiology and Pain Medicine, Anam Hospital, Korea University College of Medicine, Seoul, Republic of Korea; b AI Center, Korea University College of Medicine, Seoul, Republic of Korea; c Korea University School of Mechanical Engineering, Seoul, Republic of Korea; d Department of Obstetrics & Gynecology, Anam Hospital, Korea University College of Medicine, Seoul, Republic of Korea.

**Keywords:** hip arthroplasty, machine learning, mortality

## Abstract

We performed machine learning analysis of population data to determine the major determinants of 1-year mortality among geriatric patients undergoing hip arthroplasty. The data of 17,290 patients aged ≥ 65 years who underwent hip arthroplasty in 2019 were extracted from the Korea National Health Insurance Service claims database. With 1-year mortality as the dependent variable, random forest variable importance and Shapley Additive Explanations (SHAP) were used to identify the major predictors among 31 included predictors and their associations with mortality. The area under the curve of the random forest model was 74.6%. The top 10 predictors were age, red blood cell transfusion, male sex, dementia, low socioeconomic status, presence of solid tumors, history of congestive heart failure, history of chronic kidney disease, statin use, and history of peripheral vascular disease. Univariate analysis and SHAP analysis identified age (maximum SHAP value 0.17), red blood cell transfusion (0.10), male sex (0.09), dementia (0.04), low socioeconomic status (0.03), presence of solid tumors (0.06), history of congestive heart failure (0.04), history of chronic kidney disease (0.05), general anesthesia (0.02), and iron use (0.02) as positive contributors to model predictions of mortality. Higher SHAP values indicate greater contributions to model output relative to the baseline prediction. We constructed an effective prediction model for predicting mortality among patients undergoing hip arthroplasty. Appropriate interventions should be provided for high-risk patients (i.e., advanced age, multiple comorbidities, or anticipated transfusion needs).

## 1. Introduction

Hip fractures are one of the most common types of fractures in older adults, and the incidence increases as the population ages.^[1]^ Approximately 3,00,000 cases of hip fracture are reported annually in the United States, leading to a total cost exceeding $30 billion per year.^[2]^ In addition to the socioeconomic burden, hip fractures are associated with significant individual morbidity and mortality, with a 1-year mortality rate of 14 to 36%.^[3,4]^ Thus, it is crucial to develop a dependable postoperative mortality prediction model using risk factors for the early identification of older adults with hip fractures at high risk of mortality.

Early identification facilitates the implementation of timely interventions, thereby reducing postoperative morbidity and mortality. Previous studies have identified various physiological and pathological predictors of mortality after hip arthroplasty for hip fracture, such as advanced age, male sex, multiple comorbidities, cognitive impairment, and fracture stability.^[5,6]^ Nevertheless, the accuracy of earlier models using logistic regression for predicting mortality in high-risk patients undergoing hip arthroplasty for hip fracture has been low.^[7]^

Machine learning (ML) algorithms offer several advantages over traditional statistical methods, particularly in clinical prediction tasks involving complex and heterogeneous data. Unlike conventional models that often assume linear relationships and require manual feature selection, ML can capture nonlinear patterns, model high-order interactions between variables, and automatically identify the most predictive features. These features are particularly useful in analyzing large-scale healthcare data, which typically have intricate underlying relationships among clinical factors that cannot be easily detected through traditional approaches.^[8]^ Therefore, in this study, we aimed to establish a random forest – a type of ML technique – model using nationwide data to predict 1-year mortality after surgical treatment in older patients with hip fractures.

## 2. Materials and methods

### 2.1. Participants and variables

This retrospective cohort study was approved by the Institutional Review Board of the Korea University Anam Hospital (approval no. 2023AN0309; date of approval: 26.07.2023). The data of 17,290 patients aged ≥65 years with a diagnosis of hip fracture who underwent hip arthroplasty (bipolar hemiarthroplasty, total hip arthroplasty, or revision arthroplasty) in 2019 were extracted from the Korea National Health Insurance Service claims database. Diagnostic and procedure codes used to identify fracture-related hip arthroplasty are provided in Table S1, Supplemental Digital Content 1. There was no missing data given that these 17,290 participants were selected based on list-wise deletion. One-year mortality (yes vs no) after arthroplasty in 2019 was selected as the dependent variable. This study considered 31 independent variables identified from existing literature and available data.^[5,6,9,10]^ These variables included the following: 3 demographic/socioeconomic determinants in 2019, including age, female sex, and low socioeconomic status measured as an insurance fee of 1 (the highest socioeconomic group) to 20 (the lowest socioeconomic group); 3 anesthetic methods, such as spinal anesthesia, general anesthesia, and spinal epidural anesthesia, performed within 1 month of undergoing hip arthroplasty in 2019; 3 medications received within 1 year of undergoing hip arthroplasty, such as iron, antithrombotic agents, and statins; and 21 diseases including components of the Charlson Comorbidity Index, such as dementia and congestive heart failure. The Charlson Comorbidity Index has been developed as a weighted index to predict the risk of death within 1 year of hospitalization in patients with specific comorbid conditions.^[7]^ The medical history and medication history were screened using the ICD-10 and ATC codes, respectively (Table S1, Supplemental Digital Content 1).

### 2.2. ML analysis

The random forest algorithm was used to predict mortality and analyze the major determinants affecting mortality in patients who underwent hip arthroplasty. A random forest comprises a group of decision trees that make majority votes on the dependent variable (“bootstrap aggregation”). The number of trees was 100, the number of predictors sampled for each split was the square root of the number of all predictors, the splitting criterion was GINI, and the max depth of the tree was not predetermined. Further details have been described previously.^[11]^ The data of the 17,290 patients were divided into the training and validation sets at an 80:20 ratio (13,832 vs 3458 cases). The accuracy (the ratio of correct predictions within the dataset) and area under the curve (AUC; area under the plot of sensitivity vs 1 − specificity) of the trained models were assessed during validation. The AUC was defined as the degree of sensitivity when its threshold and specificity increased from 0 to 1.

Random forest permutation importance was used to identify the major predictors of mortality in patients who underwent hip arthroplasty. The Shapley Additive Explanation (SHAP) method, derived from cooperative game theory, was used to interpret the contribution of each variable to the model output. Each SHAP value represents the change in the predicted probability of mortality when a specific variable is included in the model compared with when it is excluded, averaged across all possible feature combinations.^[12]^ A positive SHAP value indicates that the variable contributes toward a higher model-predicted mortality risk relative to the baseline prediction, whereas a negative SHAP value indicates contribution toward a lower predicted risk. Random forest permutation importance determined the overall decrease in accuracy by permuting the data of the predictor. It was defined as the average or sum of all trees in a random forest, ranging from 0 to the number of all trees. The SHAP value of a predictor measured the difference between the probability of mortality predicted via ML with and without the predictor. For instance, the range of SHAP values for general anesthesia was (−0.01, 0.02). Some participants had SHAP values of −0.01, whereas others had SHAP values of 0.02. The inclusion of a predictor (for instance, general anesthesia) into ML altered the probability of the dependent variable (mortality) within the range of −0.01 and 0.02. A positive association between general anesthesia and mortality was assumed in this study based on univariate analysis (chi-square analysis) findings. The maximum SHAP value was used for interpretation. The inclusion of general anesthesia in ML increased the probability of the dependent variable (mortality) by 0.02. Permutation importance is considered a “global approach” as it involves determining the “average (or sum)” value for all predictions in all participants. However, SHAP can be considered a “local approach” as it involves determining the prediction for a single participant.^[13]^

Random forest permutation importance was used to derive the rankings and values of all factors in predicting the dependent variable. The SHAP plots were used to evaluate the associations (or contributions) between the predictors and dependent variables. This evaluation was performed via linear or logistic regression before developing the SHAP approach. However, unlike linear or logistic regression, the SHAP approach has a notable strength: it considers all possible scenarios. For instance, if 3 predictors of mortality – age, red blood cell (RBC) transfusion, and general anesthesia – were detected in patients who underwent hip arthroplasty, the SHAP value of general anesthesia for predicting the mortality of a particular participant was defined as the difference between the probability of mortality predicted by ML with and without general anesthesia. The SHAP value for the participant was the average of the following 4 scenarios: age excluded, RBCs excluded; age included, RBCs excluded; age excluded, RBCs included; and age included, RBCs included. Therefore, the SHAP value combined the results of all possible scenarios, unlike linear or logistic regression, in which some results are excluded owing to the unrealistic assumption of *ceteris paribus*, that is, “all other variables remain constant.” All analyses were performed using R-Studio 1.3.959 (R-Studio Inc.: Boston) between January 1st, 2024, and April 30th, 2024.

## 3. Results

Table 1 presents the descriptive statistics of the participants. The 1-year mortality rate of patients who underwent hip arthroplasty in 2019 was 13.5% (2329/17,290). The mortality rates were higher among participants with the following characteristics: advanced age, increased requirement for RBC transfusion, male sex (74.2% vs 61.2%), dementia (71.3% vs 52.2%), low socioeconomic status, presence of solid tumors (35.4% vs 22.8%), history of congestive heart failure (47.4% vs 32.8%), history of chronic kidney disease (18.5% vs 9.9%), general anesthesia (33.5% vs 31.0%), and use of iron (31.2% vs 25.0%).

**Table 1:** Descriptive statistics and variable importance of the random forest. The average feature importance was calculated as 0.024390, with a 95% confidence interval of 0.0001290 to 0.1500795.

Variable	1-yr mortality		*P*-value	VI	SHAP	
	Yes (N = 2329, 13.5%)	No (N = 14,961, 86.5%)			Max	Min
Age (mean)	83.61	78.79	.000	0.30	0.17	−0.07
Red blood bell (pack)	2.87	2.00	.000	0.11	0.10	−0.04
Female	1425 (61.2%)	11,099 (74.2%)	.000	0.08	0.09	−0.03
Dementia	1661 (71.3%)	7808 (52.2%)	.000	0.06	0.04	−0.05
Socioeconomic status (mean)	10.77	11.26	.003	0.06	0.03	−0.01
Solid tumor	824 (35.4%)	3415 (22.8%)	.000	0.05	0.06	−0.02
Congestive heart failure	1104 (47.4%)	4904 (32.8%)	.000	0.04	0.04	−0.02
Chronic kidney disease	430 (18.5%)	1486 (9.9%)	.000	0.03	0.05	−0.01
Statin	901 (38.7%)	6872 (45.9%)	.000	0.02	0.02	−0.02
Peripheral vascular disease	1397 (60.0%)	9635 (64.4%)	.000	0.02	0.02	−0.01
Cardiovascular disease	1242 (53.3%)	6623 (44.3%)	.000	0.02	0.02	−0.01
Myocardial infraction	694 (29.8%)	3430 (22.9%)	.000	0.02	0.02	−0.01
Peptic ulcer disease	644 (27.7%)	4549 (30.4%)	.008	0.02	0.01	−0.02
General anesthesia	780 (33.5%)	4645 (31.0%)	.019	0.02	0.02	−0.01
Thrombocytopenia	233 (10.0%)	884 (5.9%)	.000	0.02	0.04	0.00
Iron	726 (31.2%)	3745 (25.0%)	.000	0.02	0.02	−0.01
COPD	1188 (51.0%)	6625 (44.3%)	.000	0.01	0.01	−0.01
Tranexamic acid	682 (29.3%)	5009 (33.5%)	.000	0.01	0.01	−0.01
Anemia	1749 (75.1%)	9881 (66.0%)	.000	0.01	0.01	−0.01
Hypothyroidism	381 (16.4%)	2620 (17.5%)	.181	0.01	0.01	−0.02
Spinal anesthesia	1489 (63.9%)	9666 (64.6%)	.542	0.01	0.01	−0.01
Liver disease	1734 (74.5%)	11,110 (74.3%)	.863	0.01	0.01	−0.01
Diabetes mellitus	1729 (74.2%)	10,461 (69.9%)	.000	0.01	0.00	−0.01
Hypertension	2026 (87.0%)	12,461 (83.3%)	.000	0.01	0.01	−0.01
Hemiplegia	162 (7.0%)	1028 (6.9%)	.916	0.01	0.02	−0.02
Antithrombotic	2037 (87.5%)	13,168 (88.0%)	.466	0.01	0.01	0.00
Thyrotoxicosis, hyperthyroidism	158 (6.8%)	1032 (6.9%)	.874	0.01	0.01	−0.01
Connective tissue disease	91 (3.9%)	665 (4.4%)	.260	0.00	0.01	−0.01
Spinal epidural anesthesia	35 (1.5%)	438 (2.9%)	.000	0.00	0.00	−0.01
Lymphoma	34 (1.5%)	136 (0.9%)	.017	0.00	0.02	0.00
AIDS	3 (0.1%)	49 (0.3%)	.154	0.00	0.00	0.00
Leukemia	6 (0.3%)	28 (0.2%)	.644	0.00	0.00	0.00

Area under the curve, 0.7458.

AIDS = acquired immunodeficiency syndrome, COPD = chronic obstructive pulmonary disease, SHAP = Shapley Additive, VI = variable importance.

**Figure 1.**
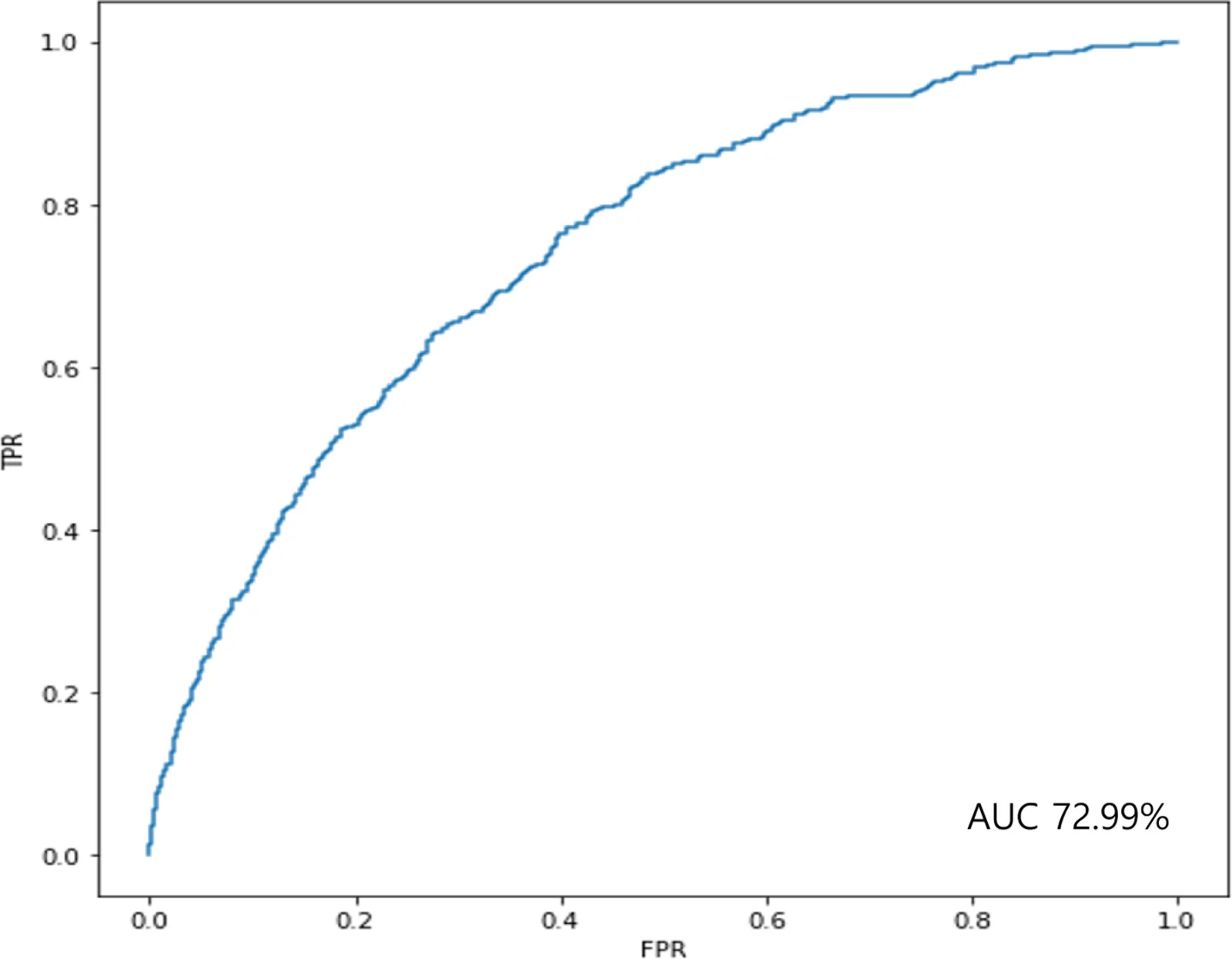
Area under the curve for the random forest model. AUC = area under the curve.

The random forest model had an accuracy of 74.6%, and the AUC for the model was 72.99% (Fig. 1). The model achieved a precision of 0.7744, recall of 0.9716, and F1-score of 0.8619 on the test set, indicating strong discriminative ability. To address the issue of generalizability, we conducted a 5-fold cross-validation using the entire dataset. The accuracy across the 5 folds was 0.7597, 0.7464, 0.7548, 0.7467, and 0.7530, with an average of 0.7521. Furthermore, we performed random splits of the dataset into training and test sets over 10 independent iterations, and the model’s accuracy was averaged across these runs. This analysis demonstrated a consistent performance with an average accuracy of 0.7577, indicating the robustness and reliability of the model.

**Figure 2.**
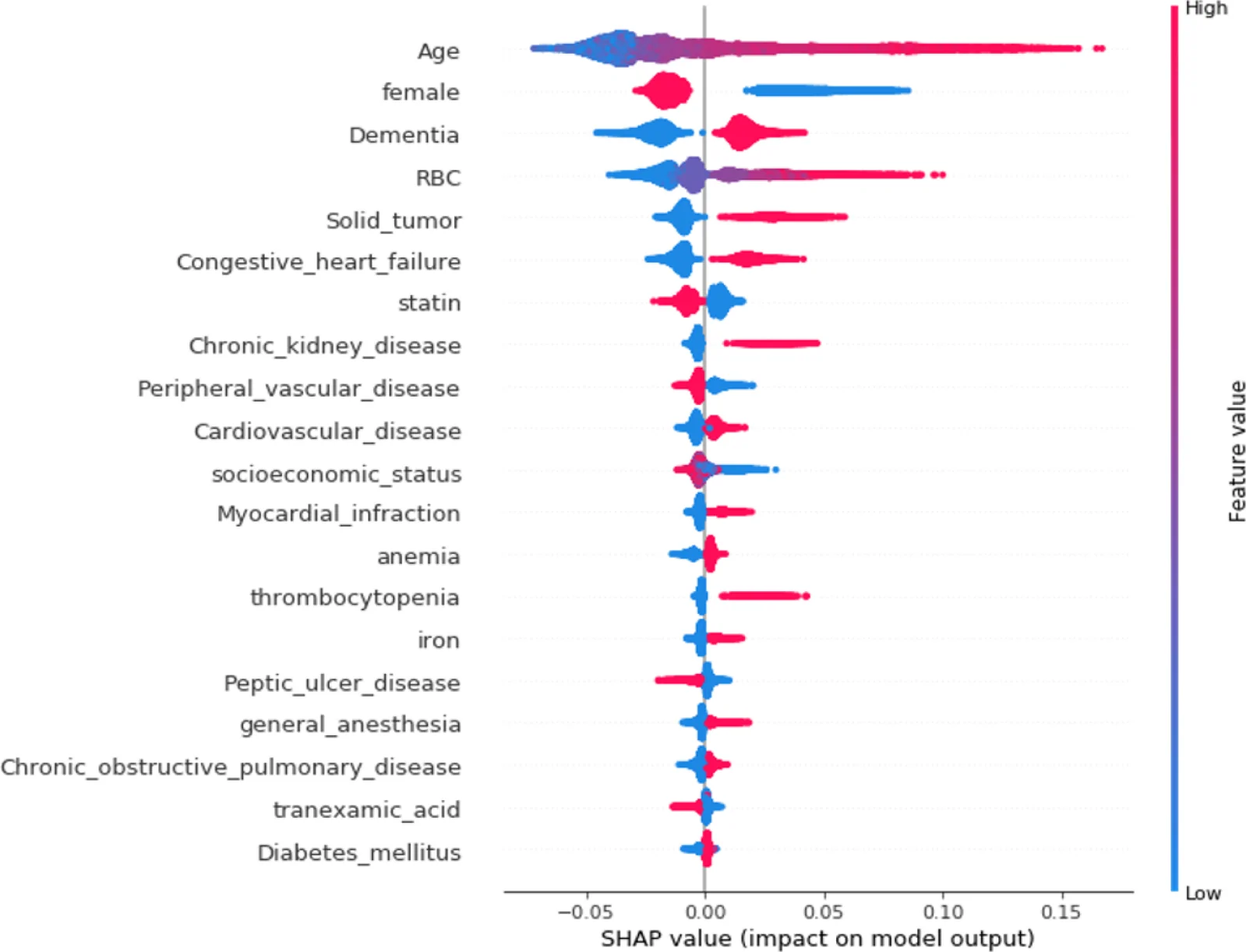
SHAP summary plot of feature importance in the random forest model. The plot visualizes the impact of each feature on the model’s prediction of 1-year mortality. Each dot represents an individual patient. The color indicates the value of the feature (red = high, blue = low), and the position along the *x*-axis reflects the SHAP value, that is, the feature’s contribution in increasing (positive SHAP) or decreasing (negative SHAP) the predicted risk. SHAP = Shapley Additive Explanation.

Age (0.30), RBC transfusion (0.11), male sex (0.08), dementia (0.06), low socioeconomic status (0.06), presence of solid tumors (0.05), history of congestive heart failure (0.04), history of chronic kidney disease (0.03), use of statins (0.02), and history of peripheral vascular disease (0.02) were identified as the top 10 mortality predictors based on the results of random forest variable importance (Table 1). The top 20 predictors included history of cardiovascular disease, history of myocardial infarction, history of peptic ulcer, general anesthesia, history of thrombocytopenia, use of iron, history of chronic obstructive pulmonary disease, use of tranexamic acid, history of anemia, and history of hypothyroidism. The results of the univariate analysis and max SHAP values (Table 1 and Fig. 2) indicated that the following predictors exhibited positive associations in general: age (0.17), RBC transfusion (0.10), male sex (0.09), dementia (0.04), low socioeconomic status (0.03), presence of solid tumors (0.06), history of congestive heart failure (0.04), history of chronic kidney disease (0.05), general anesthesia (0.02), and use of iron (0.02). Higher SHAP values indicate a greater contribution to the model output relative to the baseline prediction.

**Figure 3.**
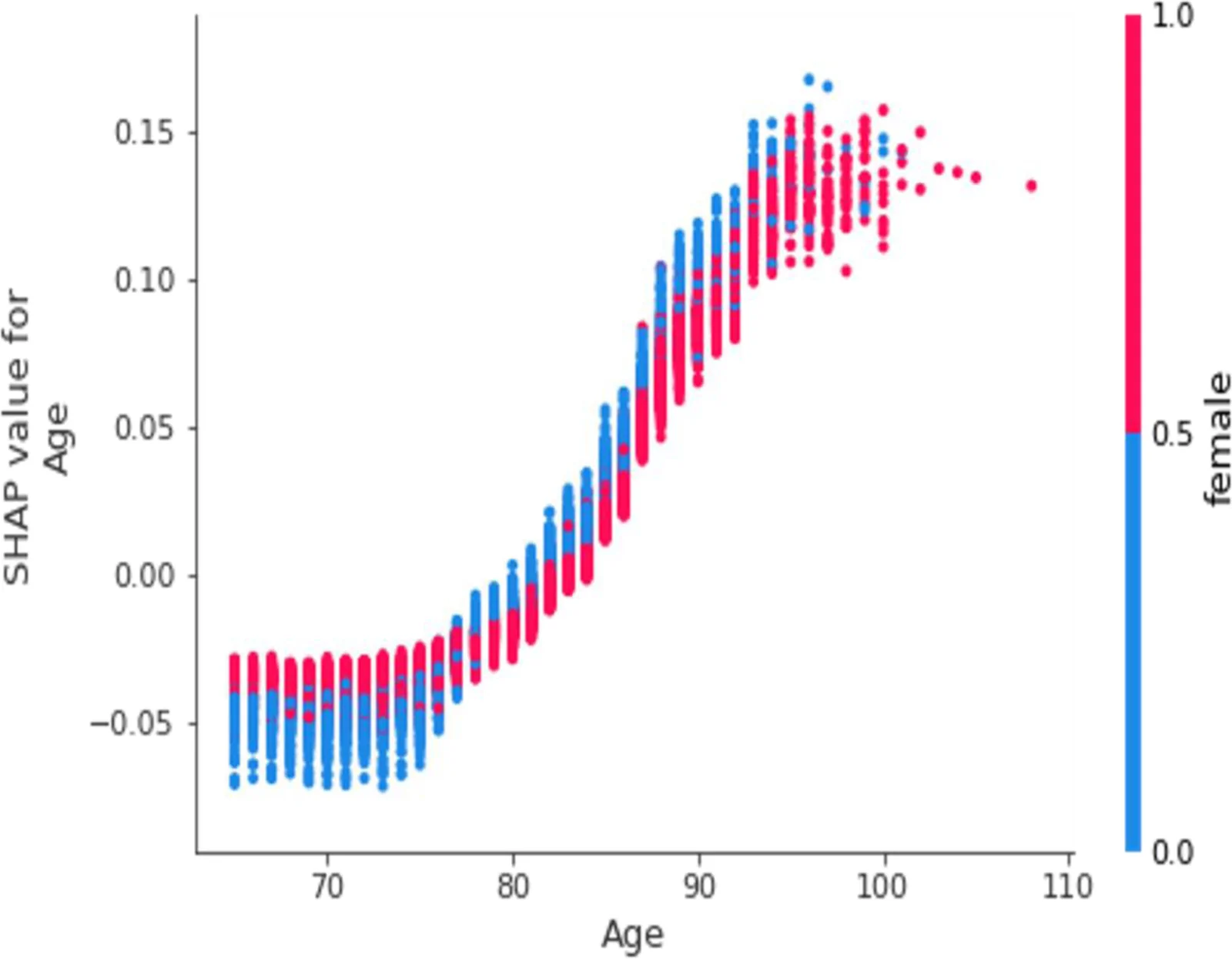
SHAP dependence plot for age in predicting 1-year mortality, colored by sex (red = female, blue = male). Each dot represents an individual, with SHAP values indicating the direction and magnitude of age’s impact. Overall, mortality risk increases with age, particularly after 85. Notably, SHAP values are higher in females until the late 70s, after which males show greater SHAP contributions. SHAP = Shapley Additive Explanation.

**Figure 4.**
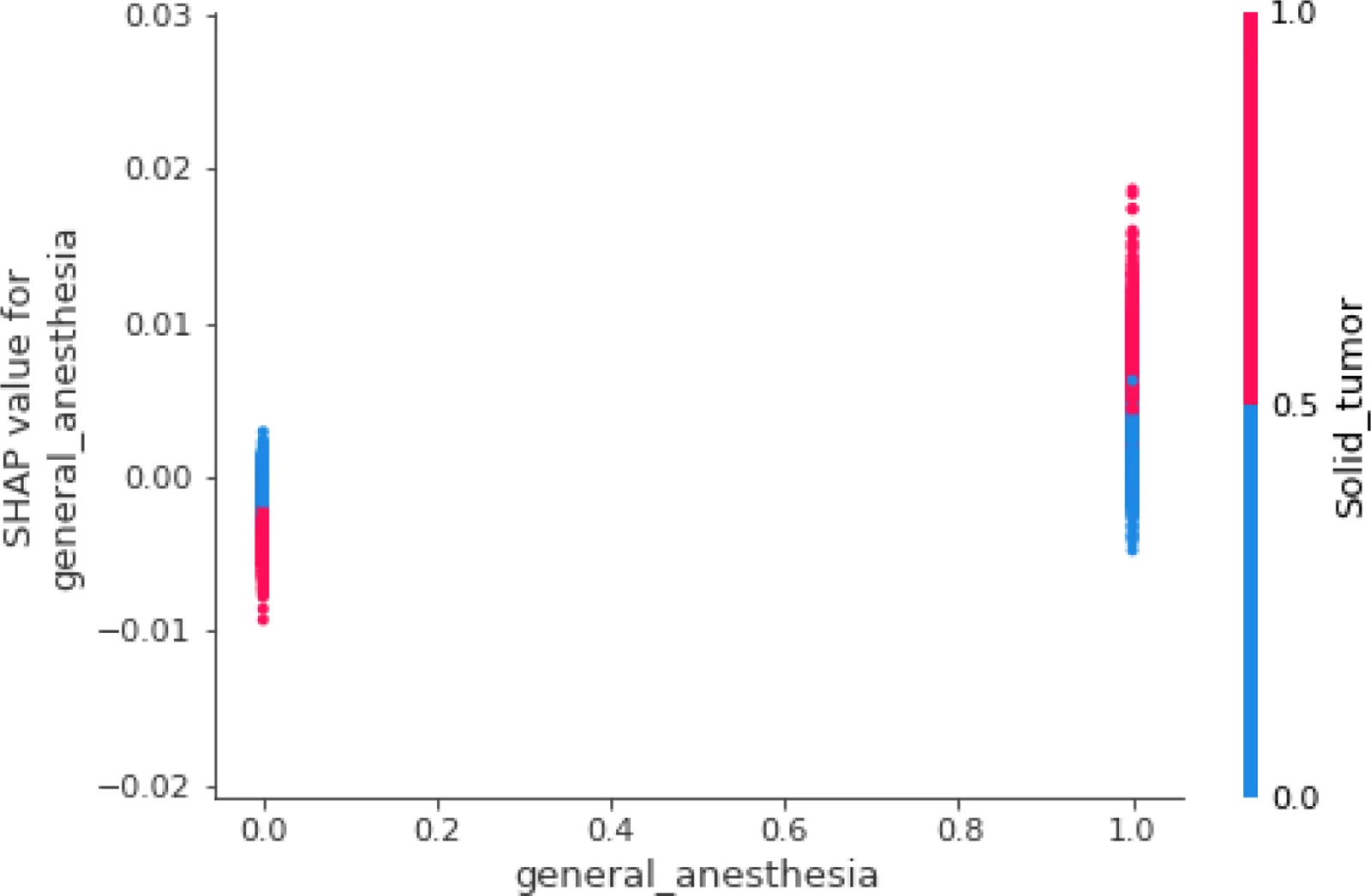
SHAP dependence plot for general anesthesia, colored by the presence of a solid tumor (red = with, blue = without). The plot shows the effect of general anesthesia on the prediction of 1-year mortality. The presence of general anesthesia (i.e., values of 1) is positioned at the top right with high SHAP values. The presence of solid tumors makes the highest contribution to the prediction of mortality with general anesthesia. SHAP = Shapley Additive Explanation.

**Figure 5.**
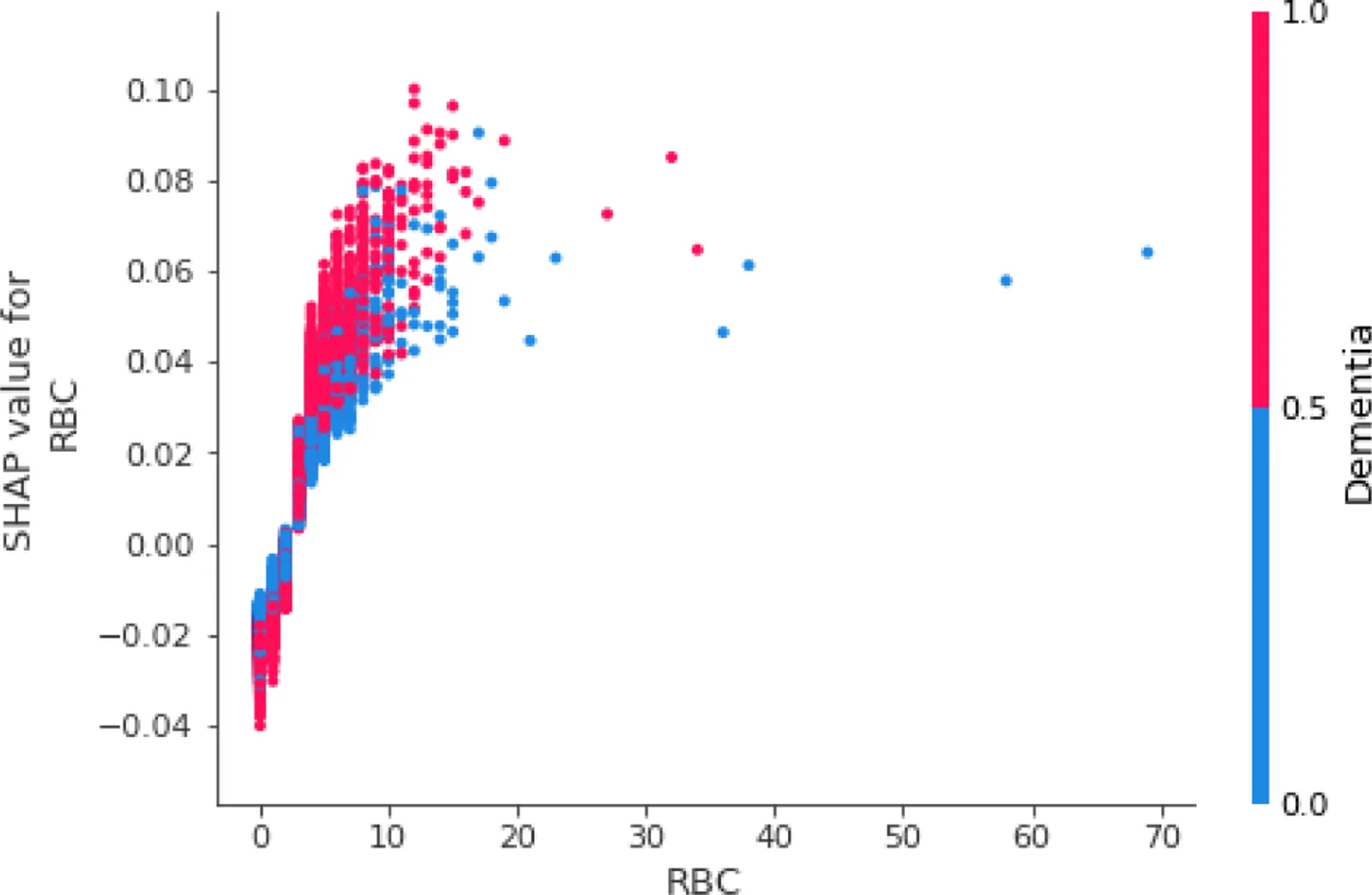
SHAP dependence plot for RBC transfusion, colored by the presence of dementia (red = with dementia, blue = without). The plot shows the impact of RBC transfusion on the prediction of 1-year mortality. The overall trend suggests that higher transfusion volumes are associated with increased predicted mortality. Notably, patients with dementia (red) tend to show higher SHAP values at similar transfusion levels, indicating a stronger influence of transfusion on mortality prediction in this subgroup. RBC = red blood cell; SHAP = Shapley Additive Explanation.

The positive association between mortality and age (a major risk factor) was more apparent in the dependence plot (Fig. 3). The horizontal and vertical axes in the figure denote age and the SHAP value of age for mortality at a particular point (i.e., a particular patient), respectively. The points with low values for age are placed at the bottom left with low SHAP values. In contrast, the points with high values for age are positioned at the top right with high SHAP values. Thus, a positive relationship was observed between age and mortality. The color blue (or red) indicates the absence (or presence) of females at a particular point. Female sex was selected as it made the highest contribution to the prediction of mortality with age. The position of the blue points (males) is above that of the red points (females) after the age of 80 years (the SHAP values of the blue points are higher than those of the red points). Similarly, the points indicating the absence of general anesthesia (i.e., values of 0) are positioned at the bottom left with low SHAP values in Figure 4. In contrast, the points indicating the presence of general anesthesia (i.e., values of 1) are positioned at the top right with high SHAP values. The color blue (or red) indicates the absence (or presence) of a solid tumor at a particular point. The presence of solid tumors made the highest contribution to the prediction of mortality with general anesthesia. The blue points indicating the absence of general anesthesia and the absence of a solid tumor are positioned at the bottom left with low SHAP values for mortality. In contrast, the red points indicating the presence of general anesthesia and the presence of a solid tumor are positioned at the top right with high SHAP values for mortality. The SHAP dependence plots for RBC transfusion and other predictors are presented in Figures 5 and S1–S29, Supplemental Digital Content 2, respectively.

## 4. Discussion

An ML model was constructed to predict 1-year post-surgery mortality in older patients undergoing hip arthroplasty by identifying the risk factors. The AUC of the model was 74.6%, and the top 10 predictors of 1-year mortality (with max SHAP value) were age (0.17), RBC transfusion (0.10), male sex (0.09), dementia (0.04), low socioeconomic status (0.03), presence of solid tumors (0.06), history of congestive heart failure (0.04), history of chronic kidney disease (0.05), statin use (0.02), and history of peripheral vascular disease (0.02). The SHAP values of these predictors are presented in Table 1 and Figure 1, which intuitively illustrate the contribution of each variable to the model prediction of 1-year mortality in older patients undergoing hip arthroplasty for hip fracture.^[12]^ Importantly, SHAP values quantify the contribution of predictors to model output relative to the baseline prediction and should not be interpreted as causal effect estimates or absolute increases in mortality risk.

Predicting mortality in older patients with hip fractures is challenging owing to the presence of comorbidities and frequent perioperative complications. The use of the random forest algorithm is advantageous in this context, as it can simultaneously handle thousands of complex clinical data points and evaluate missing data while maintaining prediction accuracy.^[4,14]^ Furthermore, it can assess the relative importance of all variables by analyzing the impact of each factor. This capability aids in the preoperative identification of high-risk older patients and the implementation of appropriate individualized interventions in advance.

To enhance clinical interpretability, we applied SHAP values to explain how individual features contribute to each patient’s predicted mortality risk. This approach helps clinicians understand the strength and direction of influence for variables such as age, comorbidities, and transfusion needs, thereby enabling preoperative risk stratification and personalized care planning. For instance, if a patient is identified as high risk based on influential features – such as advanced age, multiple comorbidities, or anticipated transfusion needs – targeted interventions such as prehabilitation, optimization of medical conditions, use of preoperative iron, or enhanced postoperative monitoring may be considered.

Further, we propose integrating the model into electronic medical records as a tool to support clinical decision-making. By inputting preoperative data, clinicians could determine the predicted 1-year mortality risk along with SHAP-based visual explanations. For instance, in a hypothetical case of a 78-year-old man with chronic kidney disease and heart failure, the model predicted a 35% mortality risk, largely driven by age and comorbidities. These insights could inform clinical decisions and promote shared decision-making.

Several studies have attempted to predict 1-year mortality using both traditional statistical methods and random forest algorithms. Reported AUC values from logistic regression–based models typically range from 0.65 to 0.75, depending on the study population and included variables.^[15,16]^ More recent ML–based models have been developed in Sweden,^[9]^ China, and Thailand^[4,7,10]^ and have demonstrated similar or slightly improved performance (AUC range: 0.72–0.78). However, many of these models were developed using smaller, single-center datasets and often lacked interpretability. In comparison, our model achieved a comparable AUC of 0.746 while offering improved interpretability using SHAP values. Additionally, the use of a large, nationally representative dataset enhances the generalizability and robustness of our findings. Together, these factors highlight the clinical relevance and potential applicability of our model in real-world settings.

Several factors, such as age, hemoglobin levels, albumin levels, parathyroid hormone levels, thyroid-stimulating hormone levels, renal failure, diabetes, and metabolic syndrome, have been identified as risk factors influencing mortality in older patients with hip fractures.^[17-22]^ In the present study, the top 10 variables associated with 1-year postoperative mortality in patients with hip fractures were age, RBC transfusion, male sex, dementia, low socioeconomic status, presence of solid tumors, history of congestive heart failure, history of chronic kidney disease, statin use, and history of peripheral vascular disease.

Advanced age and male sex are known independent risk factors for postoperative mortality in patients with hip fractures. The findings of the present study are consistent with those of previous studies.^[17-20,22,23]^

Perioperative RBC transfusion emerged as one of the strongest predictors of 1-year mortality in the present study. However, this finding should be interpreted cautiously, as RBC transfusion may influence postoperative outcomes through transfusion-related adverse effects,^[24-26]^ while also serving as a marker of underlying patient vulnerability and disease severity. Patients requiring transfusion are more likely to have preoperative anemia, greater frailty, increased surgical complexity, substantial blood loss, or perioperative physiological instability, all of which may independently contribute to adverse outcomes. Therefore, the prognostic importance of transfusion observed in our model likely reflects both the potential effects of transfusion itself and the underlying clinical conditions that necessitated transfusion.

The effect of anesthetic technique on mortality after hip arthroplasty for hip fracture remains controversial. Although some observational studies have suggested that general anesthesia may be associated with increased short-term mortality and postoperative complications compared with regional anesthesia,^[17,27]^ and general anesthesia showed a slightly higher maximum SHAP value than spinal or combined epidural–spinal anesthesia in our model, these findings should be interpreted with caution. In the REGAIN randomized trial,^[28]^ no significant differences in mortality, postoperative delirium, or functional recovery were observed between spinal and general anesthesia among patients eligible for either technique, excluding those with contraindications to spinal anesthesia such as anticoagulation therapy, severe aortic stenosis, or elevated intracranial pressure. Furthermore, anesthetic selection in routine clinical practice is often influenced by factors including hemodynamic instability, severe comorbidities, cognitive impairment, and surgical complexity. Therefore, the observed association between general anesthesia and mortality prediction may partly reflect underlying differences in patient characteristics and perioperative risk profiles.

This study has some limitations that warrant consideration. First, as our model was developed using administrative claims data, detailed clinical variables, such as laboratory results, frailty scores, and estimated intraoperative blood loss, were not available. Although transfusion emerged as a strong predictor, it may reflect residual confounding such as preoperative anemia or severe intraoperative bleeding and thus should be interpreted with caution. Second, while the model demonstrated good predictive performance, it was developed retrospectively and is not yet optimized for real-time clinical use. Additional work is required to integrate the model into electronic medical record systems and validate its feasibility in prospective, real-world settings. Finally, although the model demonstrated good predictive performance in this nationwide cohort, external validation and formal calibration assessment were not performed. Furthermore, the primary objective of this study was to identify important predictors and explore their relative contributions using ML and SHAP-based interpretation rather than to develop a clinically deployable risk prediction tool. Therefore, clinical implementation should be approached cautiously until the model has been externally validated and its calibration has been evaluated across diverse healthcare settings. Differences in coding practices, healthcare delivery systems, and perioperative management strategies may further influence model performance and transportability.

In conclusion, ML was used to construct a random forest model that could effectively predict mortality in patients undergoing hip arthroplasty. Patients in the high-risk group (age, RBC transfusion, and comorbidities) must be treated with appropriate interventions.

## Author contributions

**Conceptualization:** Kwang-Sig Lee, Hyeon Ju Shin, Ki Hoon Ahn.

**Data curation:** Hyunyoung Seong, Kwang-Sig Lee, Yumin Choi, Jeonghoon Lee, Taesan Kim, Hyeon Ju Shin.

**Formal analysis:** Yumin Choi.

**Investigation:** Hyunyoung Seong, Jeonghoon Lee, Taesan Kim, Surim Hong, Hyeon Ju Shin.

**Methodology:** Kwang-Sig Lee, Yumin Choi.

**Supervision:** Kwang-Sig Lee, Hyeon Ju Shin, Ki Hoon Ahn.

**Validation:** Hyunyoung Seong, Hyeon Ju Shin.

**Visualization:** Hyunyoung Seong, Yumin Choi.

**Writing – original draft:** Hyunyoung Seong, Kwang-Sig Lee, Yumin Choi, Surim Hong.

**Writing – review & editing:** Hyeon Ju Shin, Ki Hoon Ahn.




























































