# Supplementary material for: Explainable artificial intelligence for predicting mortality in geriatric patients undergoing hip arthroplasty: Machine learning analysis using national health insurance data
Source: Medicine (Baltimore). 2026 Sep 18;105(38):e50463. doi: 10.1097/MD.0000000000050463 (PMC13593146; doi:10.1097/MD.0000000000050463)

Supplemental Figure 2. SHAP Dependence Plot for Dementia
SHAP dependence plot showing the impact of dementia on model output, with color indicating patient sex.

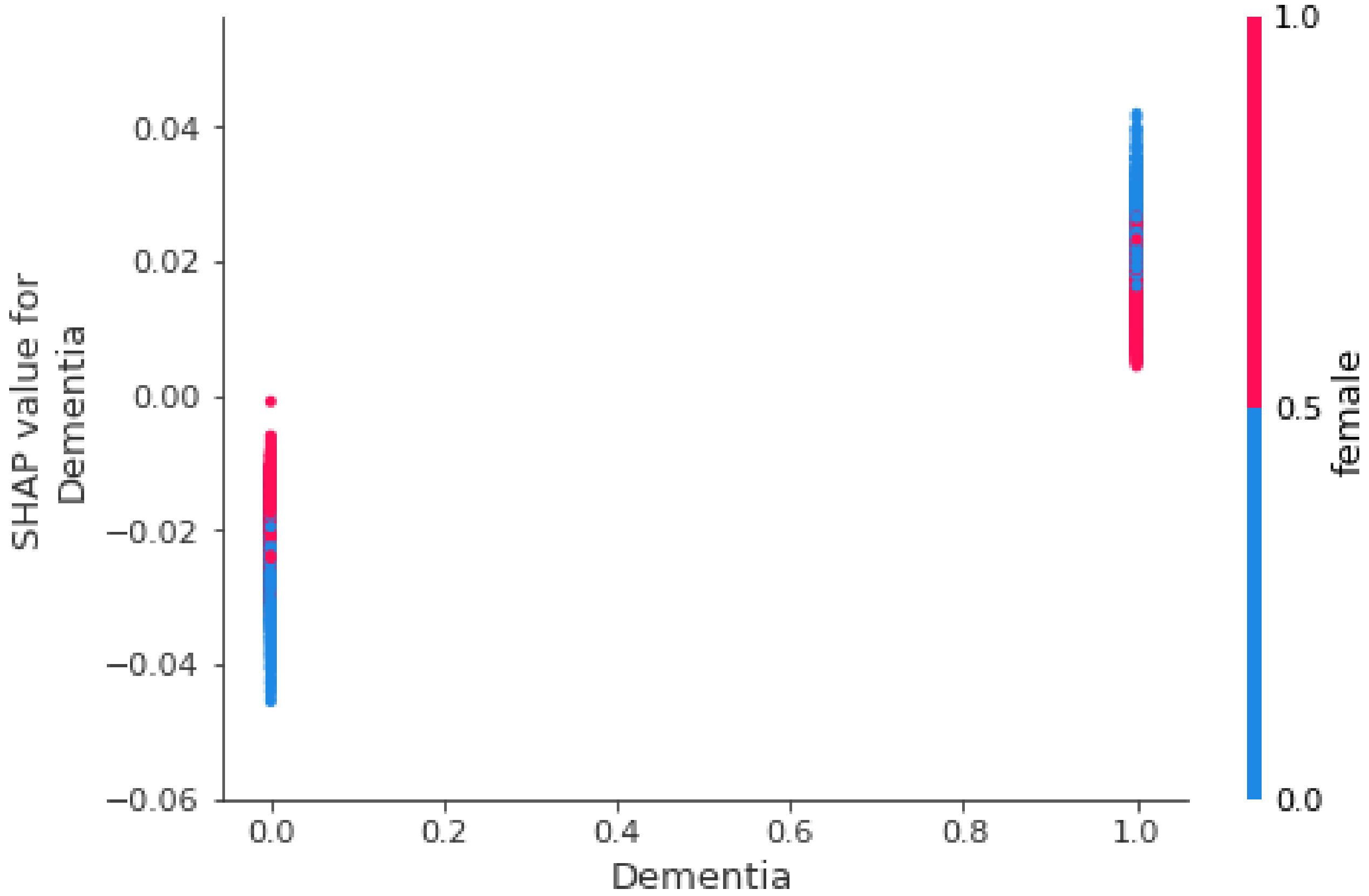

Supplement: Supplementary file 2 [file medi-105-e50463-s003.pdf]
