# Supplementary material for: Explainable artificial intelligence for predicting mortality in geriatric patients undergoing hip arthroplasty: Machine learning analysis using national health insurance data
Source: Medicine (Baltimore). 2026 Sep 18;105(38):e50463. doi: 10.1097/MD.0000000000050463 (PMC13593146; doi:10.1097/MD.0000000000050463)

Supplemental Figure 25. SHAP Dependence Plot for Connective tissue disease SHAP dependence plot showing the impact of connective tissue disease on model output, with color indicating dementia.

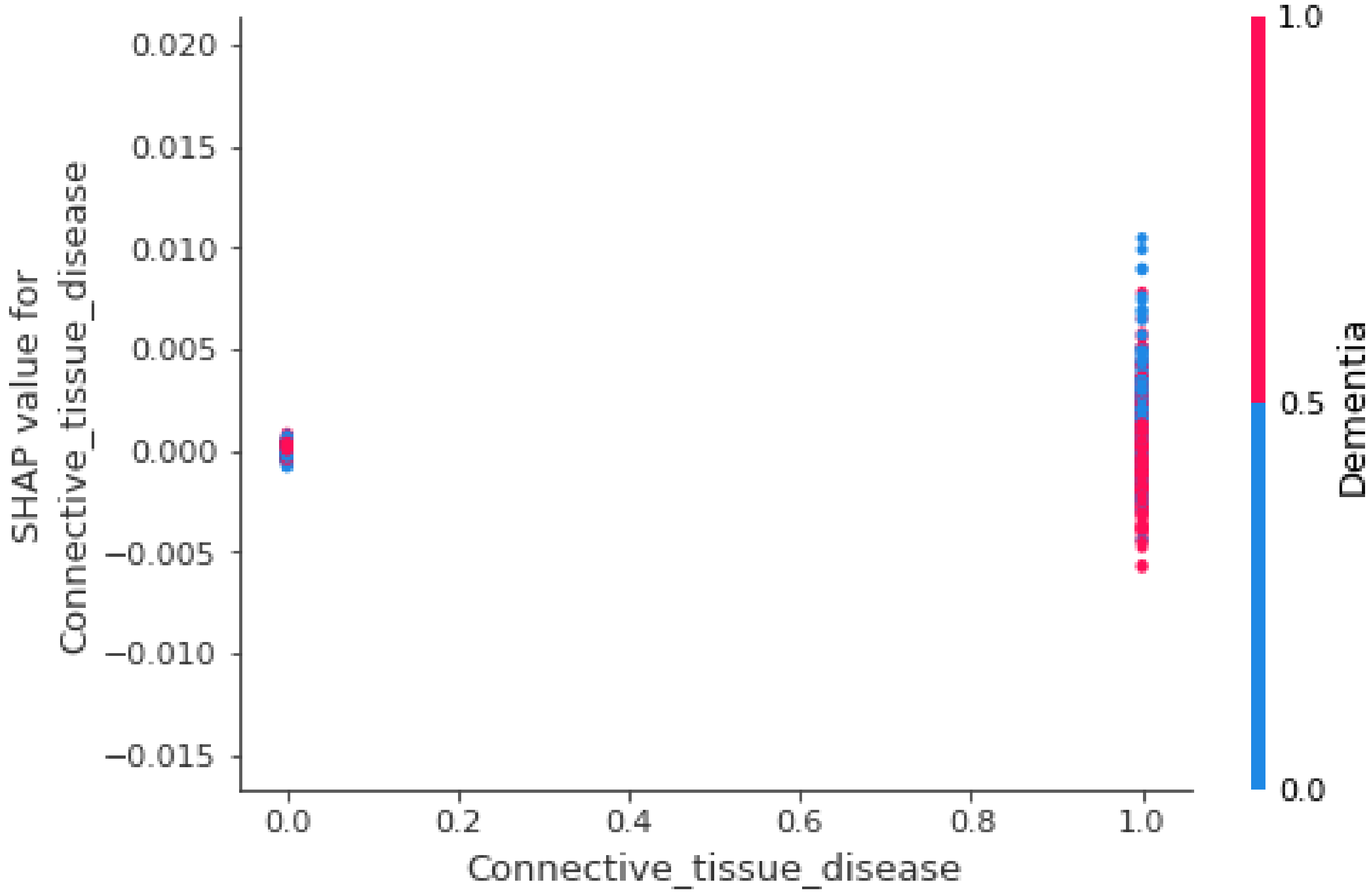

Supplement: Supplementary file 25 [file medi-105-e50463-s026.pdf]
